# Microbial profile of diabetic foot osteomyelitis from the northwest of England

**DOI:** 10.1186/s40842-024-00193-6

**Published:** 2024-11-09

**Authors:** Sara Metaoy, Iulia Rusu, Anand Pillai

**Affiliations:** 1 University of Manchester, Manchester, UK; 2 Wythenshawe Hospital Manchester, Wythenshawe, UK

## Abstract

**Background:**

Osteomyelitis of the diabetic foot is a common and challenging complication affecting patients with diabetic foot ulcers and infections. The complexity of these infections lies in their polymicrobial nature, high rates of persistence and recurrence. This study examined the microbiological profile of diabetic foot osteomyelitis from a teaching hospital in Northwest England and their resistance patterns to understand its impact on infection persistence and to direct effective treatment.

**Methods:**

A retrospective review of 105 patients who underwent surgical management for diabetic foot osteomyelitis between 2019 and 2024. We analysed three consecutive culture samples for each patient to assess for the microbiological profile and resistance patterns of these infections and to monitor infection recurrence and persistence rates.

**Results:**

A total of 105 patients were identified. Infection eradication was noted in 42% of the cohort, infection persistence in 18%, and late infection recurrence in 40%. Polymicrobial growth was evident in 72% of our study sample. Gram-positive bacteria made up the majority of the bacterial isolates in all 3 culture samples, 74.81% in sample 1, 69.31% in sample 2, and 55.1% in sample 3. *Staphylococcus aureus* was the most prevalent gram-positive bacteria, at 52.38% in sample 1, 36.19% in sample 2, and 18.09% in sample 3, followed by *Haemolytic Streptococcus, Enterococcus and Corynebacterium.* The frequently identified gram-negative bacteria were *Pseudomonas* in sample 1 (7.61%), *E. coli* and *Proteus* in sample 2 (5,71%), *Pseudomonas* and Proteus in sample 3 (2.85%). Gram-positive bacteria were resistant to penicillin and macrolides with resistance of staphylococcus aureus to clarithromycin identified among all 3 culture samples. Gram-negative bacteria were most resistant to amoxicillin. *Staphylococcus aureus* was responsible for infection persistence in most of our cohort (12/19) 63.15%. Among those patients, Staphylococcus was resistant to clarithromycin in 6 of the cases. The 5-year mortality rate for our study sample was 32.38%.

**Conclusion:**

This study highlights the prevalence of polymicrobial growth and multi-drug resistant pathogens in the scope of diabetic foot osteomyelitis. It highlights the predominance of *Staphylococcus aureus* and its resistant strains among patients affected by diabetic foot osteomyelitis in Greater Manchester.

## Introduction

Diabetes mellitus (DM) is a chronic illness affecting a significant proportion of our population today [1]. Although it is imperative to manage patients with diabetes more optimally in order to reduce the overall risk of diabetic foot ulcers (DFUs) and diabetic foot infections (DFIs) complicated by osteomyelitis, it is also pertinent for us to assess the microbial profile closely to gain an understanding of the microbial genera responsible for these infections as well as how best to manage them [1].

Diabetic foot infections are diagnosed clinically according to NICE guidelines by the presence of swelling, erythema, induration, and discharge [2]. To make a diagnosis, swabs are taken from deep wound sites/ tissue specimens and assessed for any bacterial growth [3]. Superficial DFIs can progress to deeper tissue leading to diabetic foot osteomyelitis (DFO). The diagnosis of DFO is more complex and requires assessment of the ulcer size (> 2 cm) and depth (> 3 mm) in addition to probe-to-bone testing (PTB) [4, 5].

Management of DFO comprises interventions undertaken by a multidisciplinary team, with podiatrists, endocrinologists, infectious disease specialists, and vascular and orthopaedic surgeons [6] all playing important roles in providing treatments ranging from antibiotic therapy, offloading, and foot care, to surgical interventions such as debridement and amputation [6]. The goal is to reduce surgical morbidity and more specifically amputation rates as they are related to a higher mortality rate for patients.

Most cases of diabetic foot osteomyelitis report the role of aerobic bacteria [7], with cultures displaying evidence of polymicrobial infections (up to 50%) [4, 8] rather than infection due to a single pathogen [9]. Polymicrobial infections are associated with worsened patient outcomes and increased amputation rates -Syed et al [10]. According to several studies, (Macdonald et al. 2021, Noor et al. 2015, Shettigar et al. 2018) [7, 11, 12], the most commonly identified pathogens responsible for diabetic foot infections were gram-positive [4, 13] with *Staphylococcus aureus* taking the lead [12]. The *Streptococcus* genus comes in a close second place. Other studies identified the gram-negative bacillus, *Pseudomonas*, as the most commonly isolated gram negative organism in DFIs followed by *Proteus* and *Klebsiella* [10]. A meta-analysis looking at the relationship between culture growth in diabetic foot infections and gross national income revealed that for those living in lower income countries [7], the culture predominantly included gram-negative organisms, the leading cause being *Pseudomonas* and *E.coli* [7].

Antimicrobial resistance [14] is a substantial problem in DFIs and DFO [15] due to the polymicrobial nature of the disease [10, 14], with different strains evading infection eradication through different mechanisms [16]. Peripheral arterial disease (PAD) delays the wound healing process and increases the susceptibility of patients to more aggressive infections [17]. It predisposes patients to polymicrobial growth (gram + ve, gram -ve and anaerobic bacteria) and infections by multidrug resistant pathogens [17].

Due to a high rate of infection recurrence, patients tend to have longer hospital stays which exposes them to more infections [18]. The nature of this recurrence also indicates that more than one antibiotic therapy regime is often administered to patients which increases the resistance rates of the pathogens through time [18].

The antibiotic stewardship programme is implemented worldwide for all infections indicating whether or not antibiotic administration may be needed. Taking DFUs into consideration, it is recommended to wait for culture results rather than treat empirically [18] as not all diabetic foot ulcerations may indicate an active infection.

Manchester is an urban area with a large migrant population [19]. It is expected that up to 30% of people living in Greater Manchester will develop diabetes in their lifetime [20] and thus will be predisposed to diabetic foot infections and osteomyelitis. Our study’s objectives are to assess and identify the microbial genera in relation to diabetic foot infections complicated by osteomyelitis to optimise treatment based on patient outcomes. We aim to highlight the predominance of polymicrobial growth and multi-drug resistant pathogens and their effects on infection persistence and recurrence. In accordance with previous data and literature, we hypothesised the predominance of multi-drug resistant gram positive bacteria among our cohort of patients within the Greater Manchester area. Worsened patient outcomes are expected among those experiencing frequent infections refractory to treatment necessitating prolonged treatment regimens.

## Methods

This is a retrospective observational longitudinal single centre study carried out at Wythenshawe Hospital, in the Greater Manchester area, United Kingdom. We collected data from patients who underwent surgical management for diabetic foot infections with osteomyelitis at Wythenshawe hospital between the 5 year time period of 2019 to 2024 (*n* = 105). All patients with diabetes mellitus treated for DFO (diagnosed on the basis of positive bone samples), at Wythenshawe Hospital were included. No patients were excluded from this study. Patients’ electronic medical records were accessed through use of the Hive system where age, gender, microbiology laboratory results, ABPI measurements, site of infection, duration of infection and surgical notes were reviewed. Data was then recorded on an Excel spreadsheet and analysed using the IBM SPSS system.

Patient outcomes were determined based on the latest recorded evidence of any culture samples and access of follow up notes and discharge letters. The first recorded culture sample available was labelled as culture sample number 1 with consecutive culture results labelled sample 2 and sample 3. Consecutive culture samples were analysed to assess for changes in the microbial profile and resistance and susceptibility patterns of DFO following treatment. They highlight the causative pathogens in relation to disease persistence and recurrence. The IWGDF/IDSA criteria were used to define infection persistence, recurrence, and eradication. Infection persistence was defined as the consistent presence of clinical signs of infection and microbial growth at the same wound site on review of lab results, while reinfection was defined as the presence of new signs of infection and microbial growth following a period of remittance. Clear culture samples with no evident clinical signs of infection (erythema, inflammation, discharge) represented infection eradication. Descriptive analysis was used to analyse our data expressing categorical variables, such as the microbial genera grown in culture, as numbers and percentages and p-values obtained by chi-square test for non-parametric categorical variables. Continuous data, such as age, were expressed as a mean. A p-value < 0.05 represented statistical significance with a 95% confidence interval.

## Results

Our cohort consisted of 105 patients where the mean recorded age was 63.92 (range 33–91). The majority of the cohort was male, 82 (78.09%), with only 23 (21.9%) being female. There was no statistically significant relationship between patient gender and DFO microbiology (*p* < 0.848) -chi-square test. Site of infection was recorded and was found to be keeping in with several other studies confirming that the nature of diabetic foot ulcerations and osteomyelitis start at the peripheries where peripheral arterial disease and peripheral neuropathy have the most effect. This is represented by our cohort in which 81 (77.14%) saw an effect in their forefoot while only 10 (9.52%) and 14 (13.33%) were affected in their mid and hind foot sections respectively. Surgical procedures included those requiring debridement, 49 (46.66%), and those requiring minor amputations, 45 (42.85%). Major below/above the knee amputation was observed in 7 (6.66%) patients. Multiple bone and tissue samples were taken intraoperatively with care taken to avoid cross contamination. Where possible antibiotic treatment was stopped before surgery. Majority of our cohort, 89 (84.76%), demonstrated positive bone samples confirmatory of OM. PAD was assessed through ABPI measurements, ultrasound arterial studies and duplex doppler studies. PAD was identified in 46 (43.81%) of our patients.

For the purpose of this study, we defined successful healing as full wound resolution with no evident clinical signs of infection and no bacterial growth in culture which was observed in 44 (41.9%) patients. Evidence of full wound healing represented successful treatment. Mean time to healing was 4 [1–5] months. Infection persistence was defined as consistent evidence of bacterial growth and clinical signs of infection at the same wound site even after treatment interventions with no evidence of wound healing over a 6 month period. Infection persistence was seen in 19 (18.09%) out of the 105 patients.

**Fig. 1:**
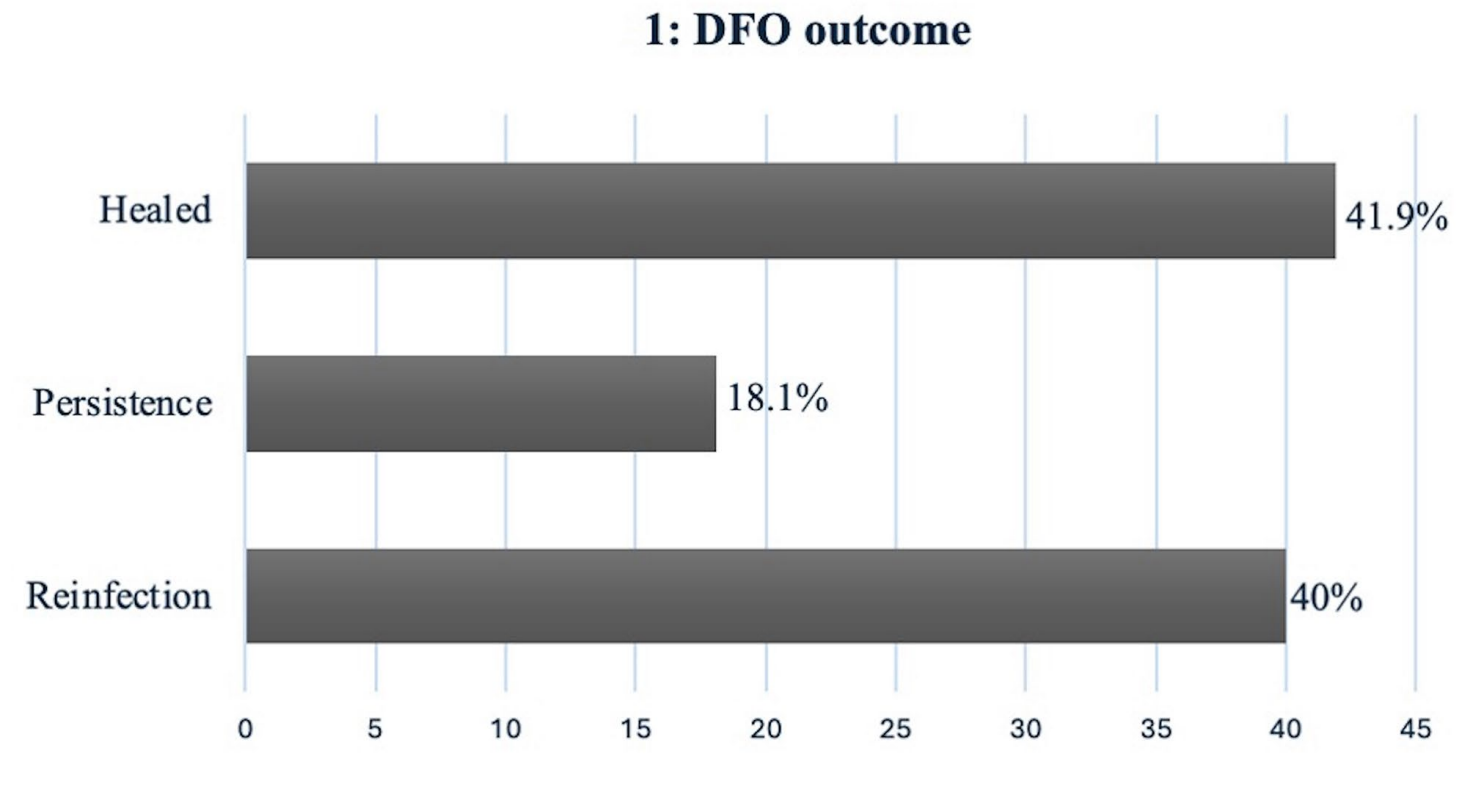
Horizontal bar chart showing the outcome of all DFO patients in our cohort

Reinfection was defined as the presence of new clinical signs of infection at a different site, or at the same site after a period of healing, with evidence of new bacterial growth. Reinfection was observed in 42 (40%) of our cohort. Mean time to reinfection was 7 (2–12) months. Patient outcomes are summarised in Fig. 1.

Out of 105 patients, 34 (32.38%) were identified as deceased. This represents the high mortality rate within DFO patients over a 5-year period of time. Chen et al. reported a higher 5 year global mortality rate of 50% in their systematic review [21].

### Microbial profile

**Fig. 2:**
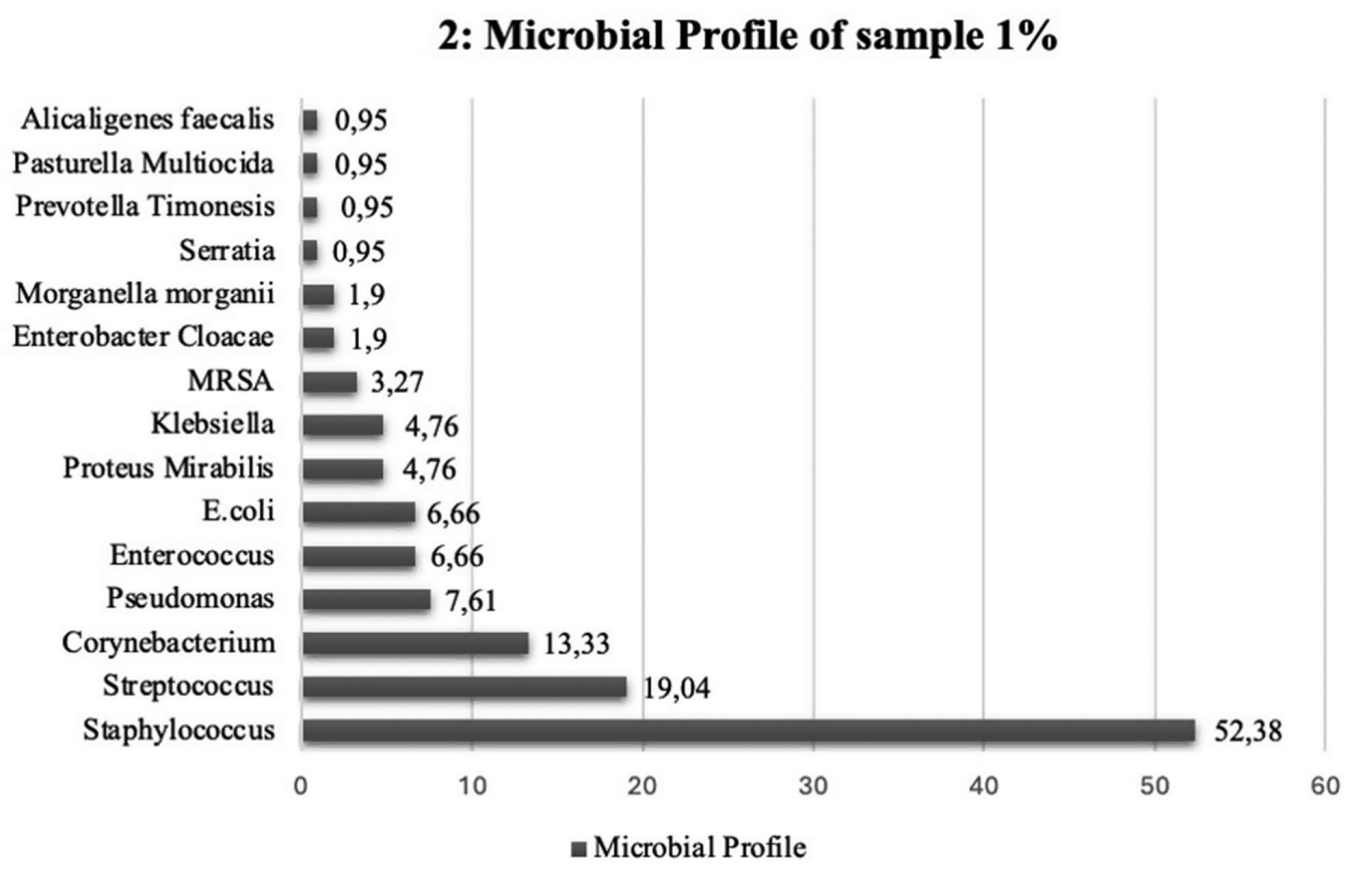
Horizontal bar chart showing culture growth from sample 1

From our cohort of 105 patients, 148 bacterial isolates were identified in sample 1. Anaerobes were identified in 12 patients, while aerobes comprised the majority with 131 identified. Among the 131 aerobes identified, 98 (74.81%) fell under the gram-positive classification with 33 (25.19%) falling under gram-negative bacteria. *Staphylococcus* was the most common bacteria identified in sample 1, 55 (52.38%). *Streptococcus* was the second most common genus affecting 20 (19.04%) of the 105 patients. The most commonly identified gram-negative pathogen was represented by *Pseudomonas* in Table 1 in 8 (7.61%) patients. The pathogens identified from our first culture sample are recorded in Table 1. and represented in Fig. 2. in the form of a horizontal bar chart.

**Table 1:** Microbial profile of DFO patients in sample 1

Variable	Number of pathogens (*n*)	Percentages (%)
Total sample	148	
Anaerobes	12	
Aerobes	131	
Gram + ve (overall)	98/131	74.81
Gram -ve (overall)	33/131	25.19
Frequency of pathogens isolated from DFO patients in sample 1		
*Staphylococcus*	55	52.38
*Streptococcus*	20	19.04
*Corynebacterium*	14	13.33
*Enterococcus*	7	6.66
*Pseudomonas*	8	7.61
*Klebsiella*	5	4.76
*Proteus mirabilis*	5	4.76
*E. coli*	7	6.66
*MRSA*	2	1.9
*Alcaligenes Faecalis*	1	0.95
*Enterobacter cloacae*	2	1.9
*Morganella morganii*	2	1.9
*Serratia*	1	0.95
*Prevotella Timonesis*	1	0.95
*Pasteurella Multiocida*	1	0.95
No pathogens isolated	5	4.76

Table 2. looks at antibiotics administered to patients according to culture growth results. Out of 105 patients, 35 (33.33%) required more than one antibiotic to help clear the infection with most needing systemic intravenous (IV) therapy later stepped down to oral therapy. Piperacillin/tazobactam (Tazocin) was administered to 33 (31.42%) of the 105 patients.

**Table 2:** Antibiotics administered in accordance with sample 1 microbiology lab results

Antibiotics administered	Frequency (*n*)	Percentages (%)
Tazocin	33	31.42
Co-amoxiclav	22	20.95
Flucloxacillin	14	13.33
Clindamycin	7	6.66
Ciprofloxacin	5	4.76
Doxycycline	5	4.76
Teicoplanin	4	3.81
Ciprofloxacin	4	3.81
Co-trimoxazole	4	3.81
Linezolid	2	1.9
Tigecycline	1	0.95
Vancomycin	1	0.95
Meropenem	1	0.95
Ceftriaxone	1	0.95
Amoxicillin	1	0.95
Metronidazole	1	0.95
Daptomycin	1	0.95

**Fig. 3:**
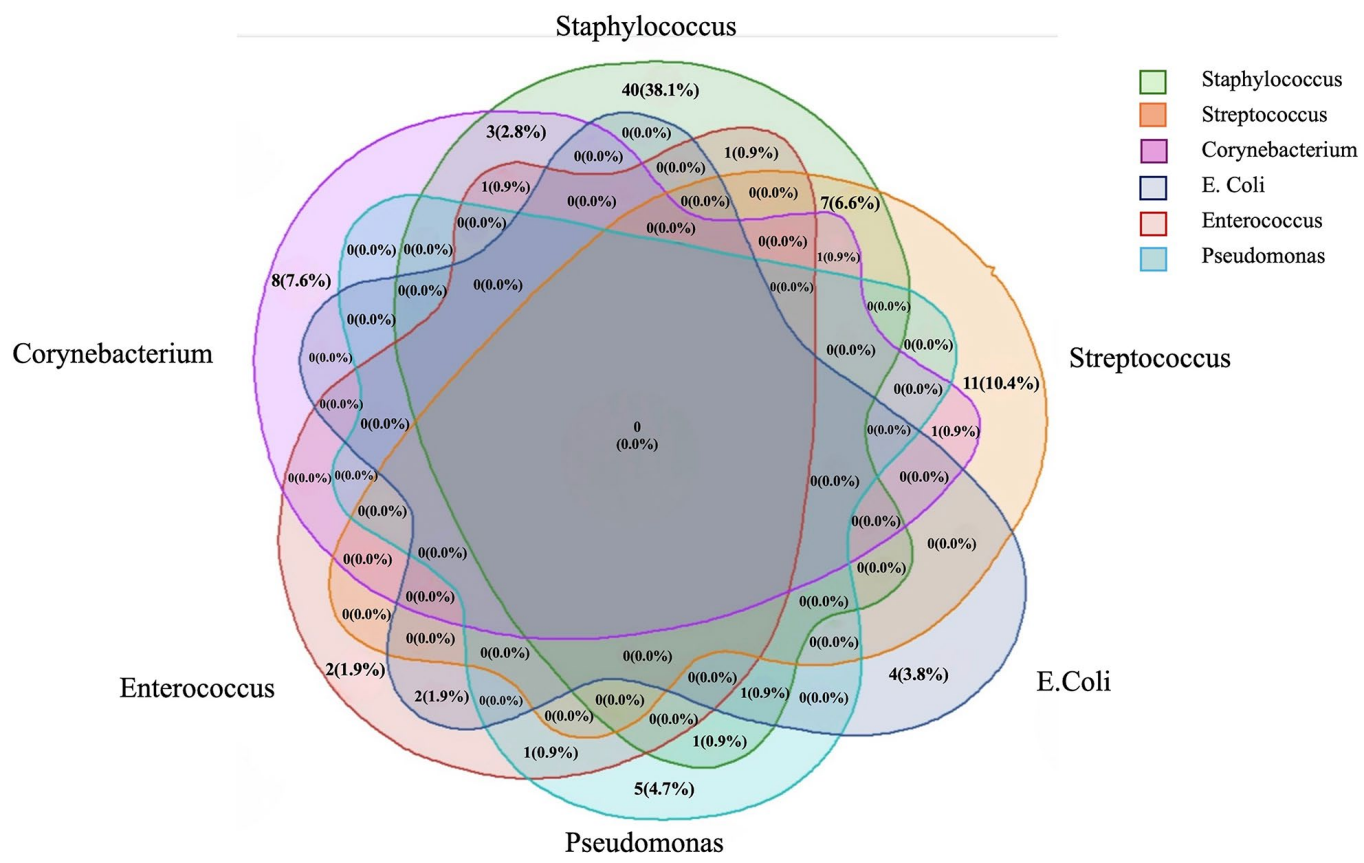
Venn diagram demonstrating bugs grown in sample 1. Bacteria with growth in more than 5 cultures are represented by this diagram, the rest were excluded

Diabetic foot osteomyelitis due to polymicrobial growth was identified in 76 (72.38%) patients. The Venn diagram in Fig. 3. represents the six pathogens identified in the majority of our cohort and draws a relationship between DFO and polymicrobial growth. Seven out of 105 patients had infections leading to the polymicrobial growth of both Streptococci and Staphylococci together, isolated from the same wound swab.

**Fig. 4:**
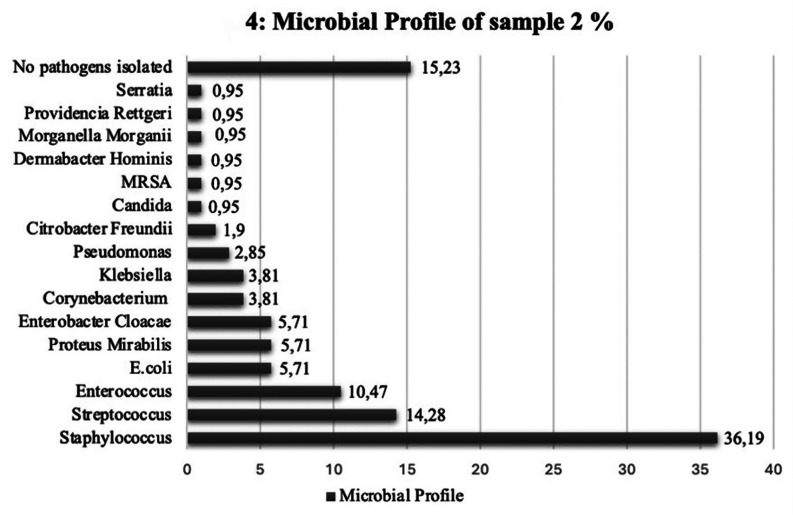
Horizontal bar chart showing culture growth from sample 2

A total of 95 antimicrobial resistance patterns were identified in sample 1. Most pathogens were resistant to clarithromycin as was seen in 22 (20.95%) patients. *Staphylococcus aureus* was resistant to gentamicin, penicillin, doxycycline, clarithromycin, clindamycin, and flucloxacillin. The second most commonly identified antibiotic resistance pattern was seen between gram-negative bacteria (*Proteus, Klebsiella*, and *E.coli*) and amoxicillin. This resistance pattern was identified in 12 (11.42%) patients.

We identified a total of 117 bacterial isolates − 16 anaerobes, 101 aerobes, 70 (69.31%) gram positive and 31 (30.69%) gram negative from sample 2. Staphylococcus was once again the most commonly grown pathogen, 38 (36.19%) -Table 3, For 16 (15.23%) patients in our cohort, no pathogens were identified from culture growth -Fig. 4. This can be noted to represent the period of temporary healing before reinfection when compared to culture growth in sample (1) Table 3, also displays the antimicrobial resistance of the bacterial genera grown in culture (2) Out of 117 identified bacterial isolates, 12 demonstrated no anti-microbial resistance profiles. The total collective antibiotic resistance patterns identified were 105.

**Table 3:** Microbial growth and antibiotic resistance profile for culture growth from sample 2, (*n* = 105)

Variable	Number of pathogens (*n*)	Percentages (%)
Total sample	117	
Anaerobes	16	
Aerobes	101	
Gram + ve (overall)	70/101	69.31
Gram -ve (overall)	31/101	30.69
Frequency of pathogens isolated from DFO patients in sample 2		
*Staphylococcus*	38	36.19
*Streptococcus*	15	14.28
*Enterococcus*	11	10.47
*Corynebacterium*	4	3.81
*Pseudomonas*	3	2.85
*E. coli*	6	5.71
*Proteus mirabilis*	6	5.71
*Candida*	1	0.95
*MRSA*	1	0.95
*Enterobacter cloacae*	6	5.71
*Dermabacter Hominis*	1	0.95
*Morganella Morganii*	1	0.95
*Citrobacter Freundii*	2	1.9
*Providencia Rettgeri*	1	0.95
*Klebsiella*	4	3.81
*Serratia*	1	0.95
No pathogens isolated	16	15.23
Frequency of antibiotic resistance patterns identified (*n* = 105)		
**Total Sample**	105	
Clarithromycin	20	19.04
Erythromycin	5	4.76
Penicillin	8	7.61
Ciprofloxacin	10	9.52
Tazocin	5	4.76
Amoxicillin	11	10.47
Clindamycin	4	3.81
Ceftazidime	1	0.95
Doxycycline	8	7.61
Flucloxacillin	3	2.85
Trimethoprim	11	10.47
Co-amoxiclav	8	7.61
Meropenem	3	2.85
Gentamicin	5	4.76
Tigecycline	1	0.95
Cefotaxime	1	0.95
Co-trimoxazole	1	0.95
*No resistance patterns identified*	12	

Clarithromycin was once again identified as the antibiotic to which most pathogens were resistant, 20 (19.04%). The second identified antibiotic resistance pattern was seen with both amoxicillin and trimethoprim represented in 11 (10.47) patients each. *Staphylococcus aureus* was resistant to clarithromycin, penicillin, erythromycin, trimethoprim, amoxicillin, and doxycycline. *Haemolytic Streptococcus* was resistant to erythromycin, clarithromycin, and doxycycline. Enterococcus faecalis was resistant to amoxicillin, flucloxacillin and clarithromycin.

**Fig. 5:**
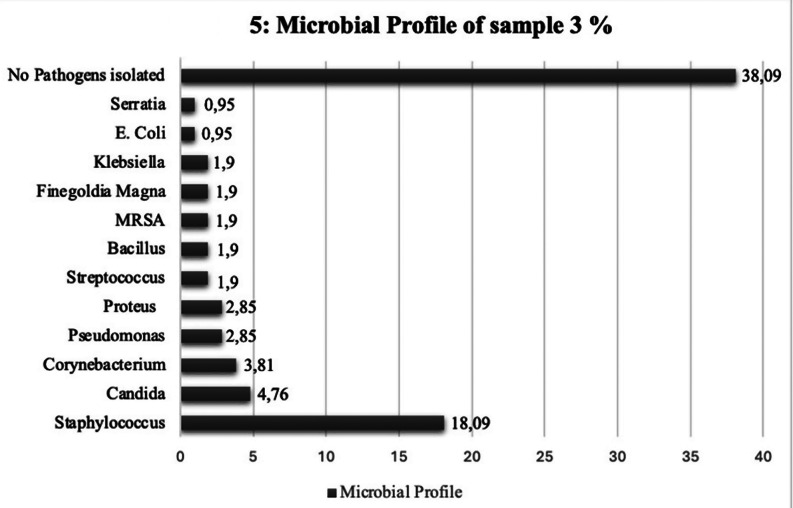
Horizontal bar chart showing culture growth from sample 3

For culture growth from sample 3, we identified a total of 49 bacterial isolates. In 40 (38.09%) patients, no pathogens were isolated which represents the cohort of patients who experienced complete infection resolution and healing. Among the bacterial isolates, *Staphylococcus aureus* was once again the most commonly identified pathogen as was represented in 19 (18.09%) patients – Fig. 5. The Antimicrobial resistance patterns identified in sample 3 were consistent with the same patterns identified in both sample 1 and 2. Staphylococcus isolates were resistant to clarithromycin, amoxicillin and flucloxacillin.

### Infection persistence

We classified infection persistence according to analysis of all available culture results over a 5 year time period. Patients with non-remitting infections displaying the same bacterial growth for 6 months in concurrent samples were classified as having persistent infections. Looking at infection persistence, 19 patients out of 105 were deemed to be affected by DFO refractory to treatment. This is represented by Table 4. We analysed 3 different culture results and discovered persistence rates most associated with either gram-positive bacteria, 16 (84.21%), or anaerobes, 3 (15.%). Staphylococcus was again identified as the main causative pathogen with 12 (63.15%) out of the 19 patients displaying infection persistence due to *S. aureus*. When investigating antimicrobial resistance, clarithromycin was the antibiotic most pathogens were resistant to, 6 (20.68%), in relation to infection persistence. This led us to conclude that a combination of infection caused by *staphylococcus aureus* with resistance to clarithromycin led to most cases of infection persistence in our study sample.

**Table 4:** Microbial growth and antibiotic resistance profile of patients exhibiting infection persistence

Variable	Number of patients (*n* = 19)	Percentages (%)
Total sample	20	
Anaerobes	20-Mar	15
Aerobes	17/20	85
Gram + ve (including MRSA)	16	84.21
Gram -ve	1	5.26
*Staphylococcus*	12	63.15
*Streptococcus*	2	10.52
*Enterococcus*	1	5.26
*MRSA*	1	5.26
*Klebsiella*	1	5.26
Frequency of antibiotic resistance patterns identified (*n* = 29)		
Clarithromycin	6	20.68
Erythromycin	4	13.79
Penicillin	4	13.79
Ciprofloxacin	3	10.34
Tazocin	2	6.89
Amoxicillin	4	13.79
Clindamycin	2	6.89
Ceftazidime	1	3.44
Doxycycline	1	3.44
Flucloxacillin	1	3.44
Trimethoprim	1	3.44

**Fig. 6:**
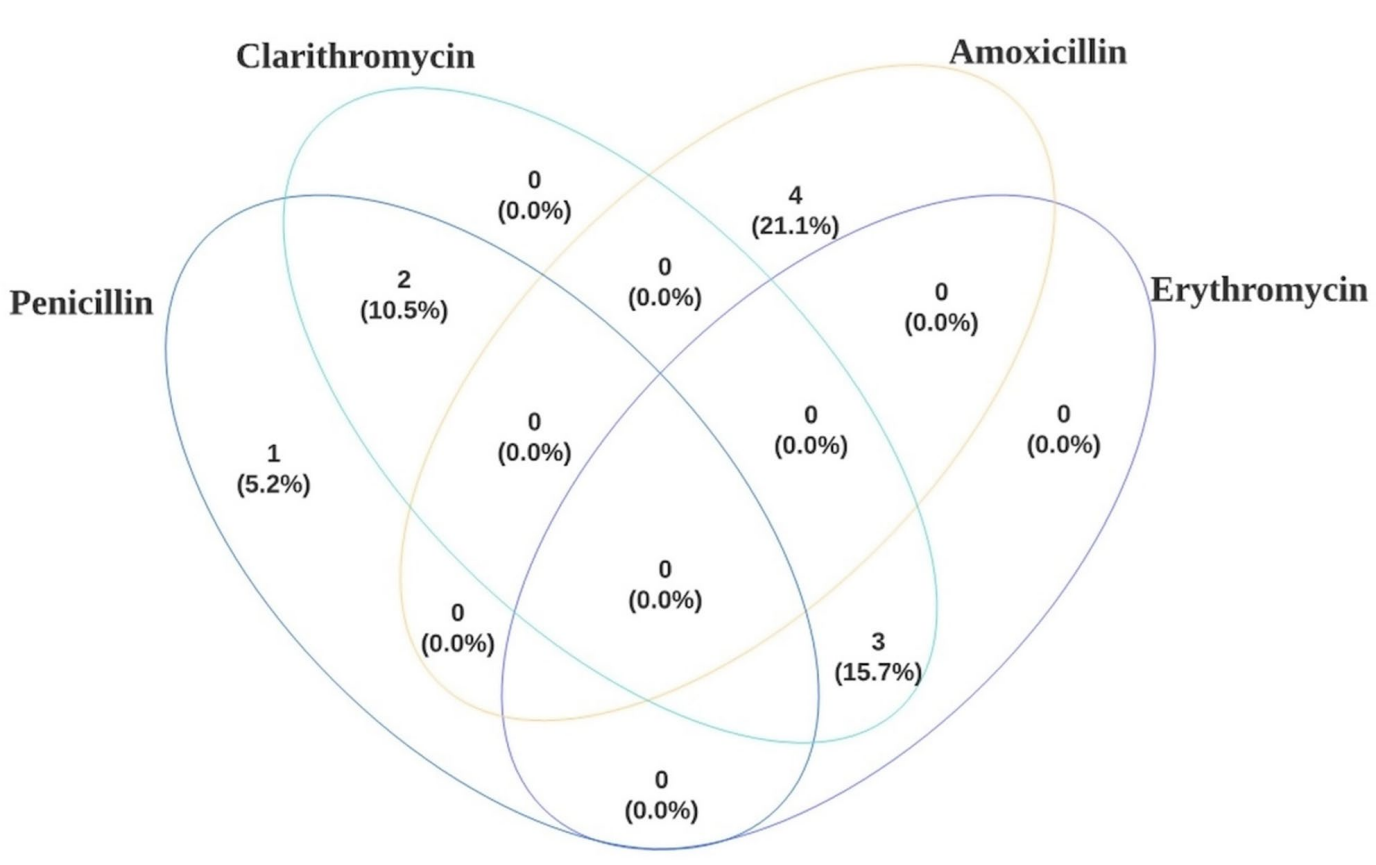
Venn diagram demonstrating antibiotic resistance profile for patients with infection persistence. Only those with 4 values or above are represented by this diagram. Overall number of antibiotic resistance profiles identified were 12

A Venn diagram representing the aforementioned antibiotic resistance profile of persistent DFO is shown in Fig. 6. All causative pathogens leading to infection persistence displayed antimicrobial resistance, demonstrating a clear relationship between infection persistence and antimicrobial resistance.

## Discussion

This study’s main aim was to determine the microbial profile of diabetic foot infections with osteomyelitis and its effect on treatment outcomes. Diabetic foot disease is a complex multifactorial complication of diabetes. The pathogenesis of DFO is difficult to fully understand and investigate which leads to a gap in the related research and literature. At the commencement of this review, the main hypothesis was that there would be a clear overruling pathogen with evident polymicrobial growth discovered on close analysis of the wound culture results. The hypothesis was that we would see gram-positive bacteria in the majority of our cohort with fewer gram-negative bacteria. This was true, on analysis of our cohort of 105 patients, gram positive bacteria were nearly threefold more likely to be responsible for diabetic foot infections. This argument was strengthened through our analysis of three different culture results obtained throughout different time frames with every culture sample favouring the growth of gram positive bacteria. The most commonly identified gram positive bacteria were *Staphylococcus aureus*, *Staphylococcus epidermidis*, *Haemolytic streptococcus*, and *Corynebacterium striatum*. Our results are comparable to those of other published studies. A study looking at the bacteriology of diabetic foot ulcers and infections [22] assessed samples taken from 102 diabetic patients with foot complications and identified *Staphylococcus aureus* as the most prevalent pathogen in diabetes-related foot pathologies (25% of all samples) [22]. Álvaro-Afonso et al. reported on the bacterial diversity in their study sample of 215 patients with DFO [15]. Their results highlighted *coagulase-negative staphylococcus*, *Staphylococcus aureus* and *Corynebacterium spp*. as the most frequently identified microorganisms [15].

Polymicrobial growth was identified in 72.38% of our patients. Similarly, a study of the microbiology of diabetic foot infections in Kuwait [23] reported a polymicrobial causes of infection in 75% of their patients [23].

A study conducted in India [24] reported gram negative bacilli as the most commonly identified pathogen in DFIs [24]. While *Pseudomonas aeruginosa* was the most commonly identified gram-negative bacteria in our cohort, it still represented a minority in relation to the total sample. This disparity between the West and the East remains a topic of discussion.

Our data revealed the overall outcome of DFO, with a high recurrence rate of 40%. Patients demonstrating full wound healing were noted to be 44 (41.9%). Time for full infection resolution and healing was 4 months. We identified the mean time frame between recurrent infections to be 7 (2–12) months. Sørensen et al. reported similar results in their study on the healing of diabetic foot ulcers in Copenhagen where the median healing time was 6 months and a 53% infection recurrence rate within 12 months [25].

The gram-positive coccus, Staphylococcus; was responsible for 12 (*n* = 19) of the cases of infection persistence. The identified resistance patterns in relation to infection persistence were observed. Among Staph. aureus isolates, the highest resistant rates were towards clarithromycin, erythromycin, and penicillin. In contrast, Dörr et al. reported the prevalence of gram-positive bacteria resistant to penicillin with low numbers of multi-resistant pathogens identified (0.9%). This discrepancy could be a result of the focus on DFUs with no account for DFO identification and diagnosis in their study [26]. A systematic review conducted in sub-Saharan Africa reported that among their study sample, *S.aureus* isolates were most commonly resistant towards gentamicin and ciprofloxacin [27].

Although there was a significant relationship between antimicrobial resistance and infection persistence, there was no significant correlation between the causative pathogen and resistance against a specific antibiotic. This was because among both patients who experienced infection recurrence and those who experienced infection persistence, the majority of the staphylococcus isolates were resistant to clarithromycin.

A high 5-year mortality rate of 32.38% was noted in our study sample. Polymicrobial growth was identified in 19/34 (55.88%) of the deceased patients, indicating a relationship between polymicrobial growth in diabetic foot osteomyelitis and poor patient outcome as highlighted by Sen and Demirdal in 2020 [28].

The prevalence of polymicrobial growth in our study and the variances between antibiotic resistance patterns within other studies aim to highlight the need for improved identification of the causative microbial genera in relation to diabetic foot infections and osteomyelitis of the diabetic foot. 16 S rRNA sequencing has in fact been a topic of discussion in recent years due to its rapid and accurate technique in identifying the aforementioned bacteria [29]. A more efficient and reliable identification of the causative pathogens and their resistance profiles could potentially lead to a reduction in inappropriate antibiotic regimens as well as an overall reduction in the progression of superficially infected wounds to osteomyelitis [29]. Our findings highlight the effectiveness of broad-spectrum antibiotics (those targeting gram-positive bacteria, Staphylococcus, and gram-negative bacteria, Pseudomonas) such as Tazocin in the Greater Manchester area for DFO following review of microbiology results.

The strengths of our study include our cohort size and identification of the microbial genera through analysis of three different culture samples. Our study is limited by its retrospective nature, our inability to access 16 S rRNA results for more accurate microbial identification as well as its focus on aerobic bacteria. Given that this is a single-centre study, our findings may not be generalisable to other populations with different demographics as well as an inherent risk of bias due to the study design.

## Conclusion

This study successfully identified the common causative pathogens in relation to DFO in the NW of England. An observed relationship between infection persistence and antimicrobial resistance, particularly staphylococcal resistance to clarithromycin was identified as the leading cause of infection persistence. Staphylococcus, more specifically *Staphylococcus aureus*, remains the main causative agent of DFO with gram-positive bacteria at the forefront. It is also the most commonly identified pathogen leading to disease recurrence. Despite advancements in wound care and antimicrobial therapy, the persistently low healing rates and high mortality incidence associated with DFO highlight the need for continued research efforts.

## Data Availability

Data is available upon request from the corresponding author.
